# A Novel Frequency-Selective Surface-Enhanced Composite Honeycomb Absorber with Excellent Microwave Absorption

**DOI:** 10.3390/polym16233312

**Published:** 2024-11-27

**Authors:** Yu-Xuan Xian, Jin-Shui Yang, Hong-Zhou Li, Chang Xu, Xiang-Wei Wang

**Affiliations:** 1 Harbin Engineering University, Harbin 150001, China; xianyuxuan@hrbeu.edu.cn (Y.-X.X.); lihz1999@hrbeu.edu.cn (H.-Z.L.); xuchang123@hrbeu.edu.cn (C.X.); wang5347@hrbeu.edu.cn (X.-W.W.); 2 Qingdao Innovation and Development Center, Harbin Engineering University, Qingdao 266000, China

**Keywords:** composite honeycomb, frequency selective surface, microwave absorption

## Abstract

Multifunctional structures with excellent wave-absorbing and load-bearing properties have attracted much attention in recent years. Unlike other wave-absorbing materials, honeycomb wave-absorbing materials have appealing radar absorption and mechanical properties. However, the existing honeycomb wave-absorbing materials have problems such as narrow absorption band and poor compression resistance. In this study, a novel frequency selective surface-enhanced composite honeycomb absorbers (FSS-CHAs) are fabricated by combining a honeycomb structure with wonderful load-bearing capacity and FSS through screen-printing and inlay-locking techniques. After reflectivity measurements, the effective absorption band (RL < −10 dB) of CHA is 6.25–17.47 GHz and a bandwidth of 11.22 GHz, the effective absorption band of the FSS-CHA is 3.96–18 GHz and a bandwidth of 14.04 GHz, 25.13% improvement compared to the CHA, the mechanism of wave absorption is explained using transmission line theory. The simulation results show that the wide bandwidth is due to the different absorption mechanisms of FSS-CHA at low and high frequencies. The compression test shows that the compression strength of FSS-CHA is 17.10 MPa. In addition, FSS-CHA has a low cost of only USD 270.7/m^2^. This study confirms the possibility of combining FSS with radar-absorbing honeycombs, which provides a reference for the design of future broadband wave-absorbing structures, offers a novel approach to integrating FSS with CHA, and aims to optimize their efficacy and utility in stealth technology.

## 1. Introduction

In recent years, the development of aircraft and ship equipment has advanced significantly [1], and how to reduce the radar cross-section (RCS) has become a hot research topic [2]. Radar absorbing materials (RAMs) play a crucial role in military applications by minimizing the risk of detection by adversaries. Besides stealth capabilities, RAMs must also fulfill additional operational requirements specific to certain scenarios [3]. Research not only focuses on the absorptive qualities of these materials but also their structural integrity—emphasizing the need for load-bearing capacity and lightweight characteristics. These factors are vital for enhancing the overall performance of military hardware [4,5,6,7,8,9]. Current studies in this field are increasingly looking towards integrated structures that combine load-bearing and absorptive functions without increasing system weight, thereby maintaining effective absorption.

Metamaterials are composite materials with extraordinary physical properties [10,11], such as electromagnetic metamaterials, mechanical metamaterials [12,13,14], and so on. Electromagnetic metamaterials have excellent absorption in a certain frequency band, such as frequency-selective surfaces (FSS). FSS is a two-dimensional structure made of a periodic arrangement of lossy dielectric patches, which has a strong absorption effect near the resonance frequency. However, FSS has poor mechanical properties, which makes the aspects of its application limited, so combining metamaterials and structures with excellent mechanical properties is expected to solve the problems and narrow absorption band of traditional radar absorbing materials. One promising structure is the honeycomb configuration, known for its exceptional mechanical properties which facilitate the use of less material while augmenting strength and minimizing weight. The development of absorption-capable, load-bearing composite honeycomb absorbers (CHAs) using honeycomb matrices shows great potential [15,16,17]. CHAs are usually categorized into flat plate and periodic three-dimensional structures [18,19]. The flat plate structures generally feature a sandwich design, comprising upper and lower skins and an aramid core. The aramid core impregnated with wave-absorbing materials acts as a radar absorber, while the upper and lower skins enhance the robustness of the structure and protect the aramid core in the center. Although flat plate structures exhibit strong bending strength, their compression resistance is relatively bad [20,21]. In contrast, three-dimensional structures are primarily based on periodicity [22,23]. The three-dimensional structure is usually a quadrilateral honeycomb with a core made of reinforced fiberglass material, and conductive materials are mounted on the walls of the honeycomb core through various techniques to ensure consistent resistance values [24,25]. The stiff honeycomb core ensures excellent compressive strength and allows performance prediction by simulation software. Kwak et al. [26] designed and fabricated a composite material consisting of a three-layer skin and a two-layer wave-absorbing honeycomb core, which achieved effective absorption from 5.8 to16.3 GHz at a height of 9 mm. Wang et al. [27] used a stitching technique to install a gradient resistive film on a glass fiber plate as a way to improve impedance matching. The experimental results show that this tetragonal honeycomb can achieve an effective absorption of 6–18 GHz at a height of 5 mm, in addition to the remarkable mechanical properties of this structure. Zhang et al. [28] prepared impedance-type square lattice composite structures (SLCSs) using glass fiber reinforced polyethylene (GFRP) with vacuum bagging technique and installed resistive films inside the ISLCSs. The experimental results show that the ISLCSs have an effective absorption band of 5.4–18 GHz, a height of 10 mm and a compressive strength of 24 MPa. Liu et al. [29] used a screen-printing technique to install a resistive film inside a honeycomb of PMI-reinforced quartz fibers, which can achieve an absorption bandwidth of 34.8 GHz (2.6–4 GHz, 6.6–40 GHz) with a compressive strength of 5.8 MPa at a thickness of 22 mm. But there are still challenges such as narrow absorption range and large thickness. At the same time, absorbing-carrying integrated structures usually lack sufficient mechanical properties, which limits the scope of use.

In this study, we designed, fabricated, and tested the performance of an FSS-enhanced composite honeycomb absorber (FSS-CHA). We used CST Studio Suite 2022 for structural design and optimization, explained the principles of electromagnetic wave absorption using the transmission line method and the equivalent circuit method, investigated the effects of various parameters on the absorptive capacity of the FSS-CHA, and explored the absorption mechanisms at low and high frequencies. The innovative combination of FSS and CHA can significantly improve radar absorption, providing a promising approach for future research.

## 2. Electromagnetic Wave Absorption Theory

For a general wave absorbing structure, the absorption rate *A* can be expressed as follows:

(1) $$A=1-{\left|S_{11}\right|}^{2}-{\left|S_{21}\right|}^{2}$$

In Equation (1), *S*_11_ is the reflectance and *S*_21_ is the transmittance, because the carbon fiber bottom plate has the effect of total reflection, so the transmittance *S*_21_ = 0, so there is Equation (2).

(2) $$A=1-{\left|S_{11}\right|}^{2}$$

(3) $$S_{11}=\frac{Z_{in}-Z_{0}}{Z_{in}+Z_{0}}$$

where *Z_in_* is the input impedance and *Z*_0_ is the characteristic impedance of the free space, generally taken as *Z*_0_ = 377 Ω. According to Equations (1) and (2), an increase in *A* requires a decrease in *S*_11_, i.e., *Z_in_* = *Z*_0_, which is the ideal perfect impedance match.

According to transmission line theory, the input impedance *Z_in_* is calculated as follows:

(4) $$Z_{in}=Z_{0}\frac{Z_{0}\tan h(d\gamma )+Z}{Z\tan h(d\gamma )+Z_{0}}$$

In the formula, *Z* is the characteristic impedance of the material, which is determined by the nature of the material itself, *d* represents the thickness of the material, and *γ* is the propagation constant. *Z*, *γ* is denoted as follows:

(5) $$Z=Z_{0}\sqrt{\frac{\mu_{r}}{\varepsilon_{r}}}$$

(6) γ=α+jβ

where *μ_r_* and *ε_r_* are the relative magnetic permeability and relative permittivity of the material, respectively, *α* and *β* are the decay and phase constants. For this structure, *α* = 0, then *γ*:

(7) $$\gamma =j\frac{2\pi f}{c}\sqrt{\mu_{r}\varepsilon_{r}}$$

**Figure 1:**
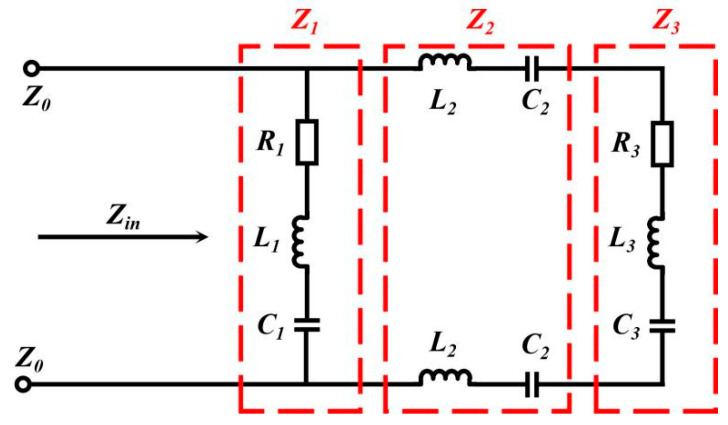
Equivalent circuit diagram of FSS-CHA.

For multilayer absorbers, the transmission line method is usually used for simulation and calculation, which is adopted for FSS-CHA, and the equivalent circuit diagram of FSS-CHA is shown in Figure 1, In this study, CHA is considered as lossy homogeneous media. The impedance of FSS, PTFE dielectric layer, and CHA are *Z*_1_, *Z*_2_, *Z*_3_.

The input impedance of each layer of a multilayer material is determined by the input impedance of the previous layer and the thickness of this layer, then *Z_in_*_1_, *Z_in_*_2_, *Z_in_*_3_ can be expressed as follows:

(8) $$Z_{in1}=Z_{1}\tanh(d_{1}\gamma_{1})$$

(9) $$Z_{in2}=Z_{2}\frac{Z_{2}\tanh(d_{2}\gamma_{2})+Z_{in1}}{Z_{in1}\tanh(d_{2}\gamma_{2})+Z_{2}}$$

(10) $$Z_{in3}=Z_{3}\frac{Z_{3}\tanh(d_{3}\gamma_{3})+Z_{in2}}{Z_{in2}\tanh(d_{3}\gamma_{3})+Z_{3}}$$

In the homogenization process of FSS-CHA, according to the Hashin–Shtrikman theory [30], the magnetic permeability *μ* and the dielectric constant *ε* of the equivalent material are, respectively, as follows:

(11) $$\mu =g\mu_{1}+(1-g)\mu_{0}$$

(12) $$\varepsilon =g\varepsilon_{1}+(1-g)\varepsilon_{0}$$

(13) $$g=\frac{a^{2}}{p^{2}}$$

(14) $${\varepsilon_{A}}^{\prime }={\varepsilon_{i}}^{\prime }\frac{(1+g){\varepsilon_{0}}^{\prime }+(1-g){\varepsilon_{i}}^{\prime }}{(1-g){\varepsilon_{0}}^{\prime }+(1+g)\varepsilon_{i}^{\prime }}$$

(15) $${\varepsilon_{A}}^{\prime \prime }={\varepsilon_{i}}^{\prime \prime }\frac{(1+g){\varepsilon_{0}}^{\prime \prime }+(1-g){\varepsilon_{i}}^{\prime \prime }}{(1-g){\varepsilon_{0}}^{\prime \prime }+(1+g){\varepsilon_{i}}^{\prime \prime }}$$

where *μ*_1_ and *ε*_1_ are the magnetic permeability and the dielectric constant of the wave-absorbing material, *μ*_0_ and *ε*_0_ are the magnetic permeability and the dielectric constant of the base material, *g* is the proportion of volume occupied by the wave-absorbing material in the vertical direction, *ε_A_*′ is the real part of the dielectric constant of the material after homogenization, *ε_A_*″ is the imaginary part of the dielectric constant of the material after homogenization. The wave-absorbing material of FSS-CHA is composed of conductive carbon ink, and the loss comes from a single dielectric loss, so the permeability *μ* = 0, therefore it is only necessary to optimize the dielectric constant to achieve better impedance matching and go for better absorption.

## 3. Design and Optimization of FSS-CHA Structure

**Figure 2:**
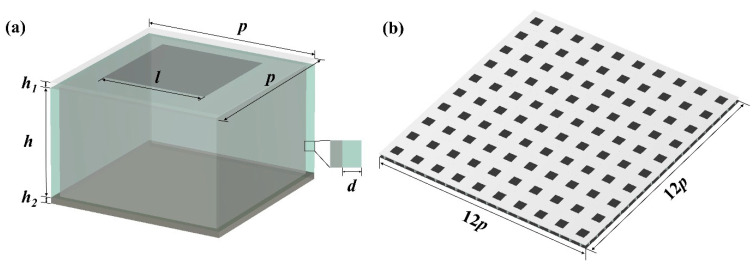
Geometric modelling of FSS-CHA. (**a**) Single FSS-CHA unit, (**b**) FSS-CHA array (180 mm × 180 mm).

During the structural design and parameter optimization, CST microwave studio is used for modeling and simulation. The geometric model of the FSS-CHA is shown in Figure 2.

The FSS-CHA structure is divided into three layers. The first layer is the FSS, which consists of periodic resistor sheets mounted on polytetrafluoroethylene (PTFE). The thickness of PTFE is *h*_1_ = 0.5 mm. The length and width of the resistor sheet is *l*. The second layer is the CHA, which is a tetragonal honeycomb made of FR-4 embedded locks with conductive ink mounted on the honeycomb walls, and is the main microwave absorbing and carrying structure. FR-4 grille length and width are *p*, wall thickness *d*, height *h*, grid wall resistance *r*_1_, and the resistance of a single resistor sheet in the FSS is *r_f_*. The third layer is made of four layers of carbon fiber T700 unidirectional prepreg pressed into the bottom plate, with a thickness of *h*_2_ = 0.4 mm, playing a full reflection of electromagnetic waves and the role of mechanical load bearing.

For the FSS in FSS-CHA, varying *l* and *r_f_* can adjust the homogenized dielectric constant of the FSS, and for the CHA, varying *p*, *d*, *h*, *r*_1_ can adjust the homogenized dielectric constant of the CHA, thus improving the impedance matching effect. The CST EM studio is next used to study the effect of each parameter on the absorption effect.

**Figure 3:**
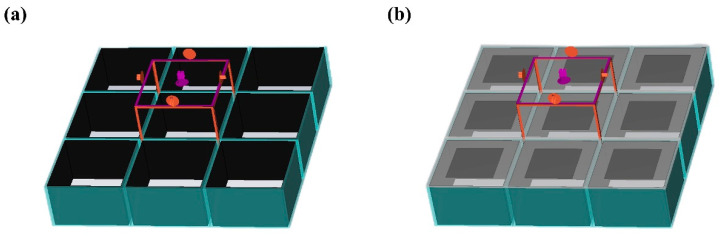
Geometric modelling in CST (**a**) CHA, (**b**) FSS-CHA.

The boundary condition in the *x*, y direction is set to unit cell to simulate an infinite single cell, the *Z_min_* direction is set to *E_t_* = 0 to simulate an all-reflective PEC base plate, and the *Z_max_* direction is set to open (add space). The finite element simulation model in CST is shown in Figure 3.

**Figure 4:**
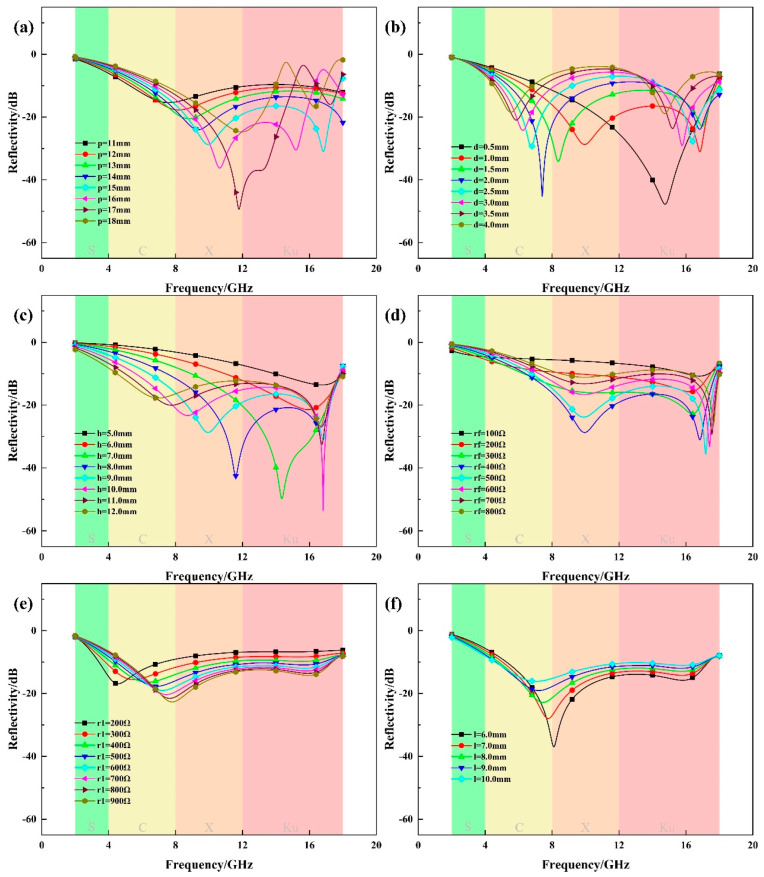
Parameter Optimization for FSS-CHA. (**a**) *p*, (**b**) *d*, (**c**) *h*, (**d**) *r_f_*, (**e**) *r*_1_, (**f**) *l*.

FSS-CHA are periodic structures that can be used to improve wave-absorbing properties by tuning the geometrical parameters of the single cell. The optimal single-cell geometric parameters that can achieve the maximum absorption bandwidth are searched by the control–variable method. We use the Par. Sweep function in the simulation module of CST to establish a task, then set up the parameters to be optimized. For example, in Par. Sweep, we set the target parameters as *p*, *h*, *d*, *r_f_*, with the ranges of 1–50, 1–20, 0.1–5, 0–1000, and the Step widths of 1, 1, 0.1, and 10, respectively. The optimization of the four parameters of *p*, *h*, *d*, and *r_f_* will require the computation of 50 × 20 × 50 × 1000 = 5 × 10^7^ algorithms. This poses a significant challenge to the server’s computing power and also results in “wasted computation”, if *p* = 1 is not effective, then all computations under the condition of *p* = 1 are meaningless. Therefore, in this paper, a control–variable approach is used in the optimization process, in which some parameters are determined first, and then subsequent parameters are determined according to these parameters to improve computational efficiency. Figure 4 exemplifies some of the optimized parameters.

First, we determine *p* in CHA when *h* and *d* is 9 mm and 1 mm, respectively. As *p* increases, the absorption effect of low frequency decreases, the absorption effect of high frequency increases, and the overall absorption effect increases first and then decreases; it is better to take *p* = 15 mm.

The thickness *d* of the honeycomb wall also plays an important role in the absorption effect. We specify *p* = 15 mm, *h* = 9 mm, and change the parameter *d*. As shown in the figure, as *d* increases, the absorption interval moves to the lower frequency, but the overall absorption interval decreases and considering various factors, *d* = 1 mm is taken. Under the condition that *p* and *d* are unchanged, increasing *h* can increase the absorption bandwidth, but the absorption effect in the low-frequency region will be reduced. At the same time, if the height is too large, it will lead to the narrowing of the application scenario, taking *h* = 9 mm.

Figure 4d shows the effect of the honeycomb wall resistance *r_f_* on the absorption performance. Increasing the resistance causes the absorption interval to first increase and then decrease, taking *r_f_* = 400 Ω. After determining the parameters of CHA, it is necessary to determine the parameters of FSS, *r*_1_ and *l.* Increasing *r*_1_ has less effect on the absorption performance, taking *r*_1_ = 600 Ω, *l* has a greater effect on the absorption performance, and increasing *l* increases the absorption bandwidth but decreases the peak, and for a comprehensive consideration, taking *l* = 9 mm. Table 1 shows the parameters in the FSS-CHA.

## 4. Experimental Section

### 4.1. Materials

FR-4 is purchased from Hongda insulation materials factory, Shenzhen, China. Conductive carbon ink (Type: CAPITON-900B-40, CAPITON-902B-4K) is purchased from Shenzhen Capiton Sci-Technology Co., Ltd., Shenzhen, China. Epoxy resin glue (Type: DP460) are provided by 3M China Limited, Shanghai, China. Polyimide (PI) film purchased from Shenzhen Brand Adhesive Co., Ltd., Shenzhen, China.

### 4.2. Fabrication

The fabrication of the FSS commenced with the application of silk-screen printing techniques to deposit conductive carbon ink onto a layer of PI film with a thickness of 0.075 mm. The resistivity of the carbon ink is adjusted using various ratios of CAPITON-900B-40 and CAPITON-902B-4K as delineated in Section 4.1. Following drying, the resistance is measured using a four-point probe resist meter until the ink mixture achieved the desired resistance ratio. Subsequently, it is adhered to a PTFE sheet.

**Figure 5:**
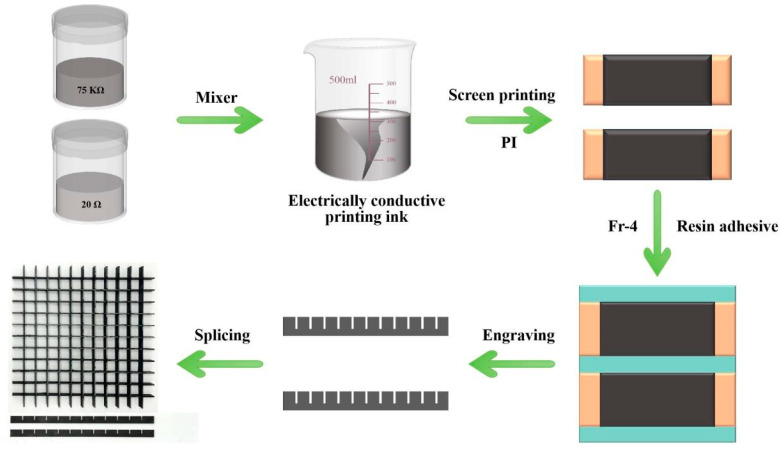
The process of fabricating CHA.

**Figure 6:**
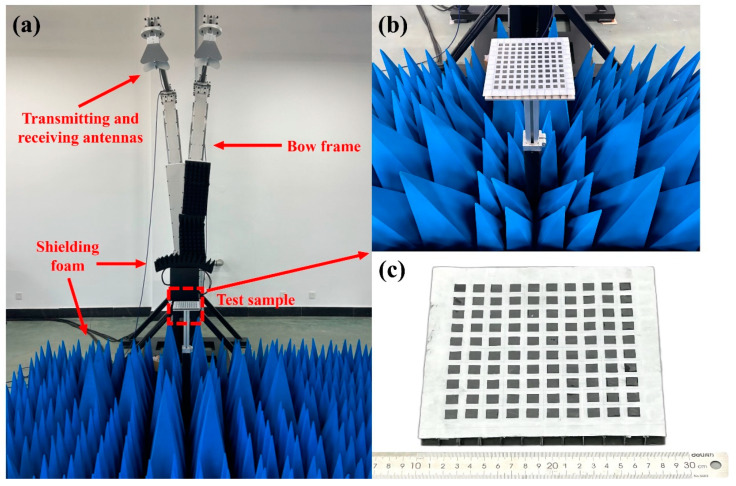
Electromagnetic wave absorption test. (**a**) Bow frame Method Test System, and (**b**,**c**) photos of the samples tested (180 mm × 180 mm).

The fabrication steps for CHA involve first allowing the resistive film to dry, followed by adhering it to an FR-4 board, using epoxy resin adhesive. After waiting for the glue to dry, the FR-4 is cut into rectangular blocks using a CNC machine and engraved with grooves as shown in Figure 5. These rectangular blocks are then inlaid and locked to assemble a lattice structure. Finally, the FSS is combined with the lattice structure. Carbon fiber board plays a role in the full reflection of electromagnetic waves. It is made by alternating four layers of unidirectional T700 prepreg, using a vulcanizing machine at 70 °C insulation for 40 min, and then warmed to 120 °C insulation for 90 min. Finally, we use epoxy resin adhesive DP460 to attach the FSS, CHA, and carbon fiber bottom plate sequentially, and cure it at room temperature for 48 h. The fabricated FSS-CHA are shown in Figure 6c.

## 5. Results and Discussion

### 5.1. Electromagnetic Properties

Experimental measurements of reflection loss are carried out in a wave-absorbing darkroom, using the bow-frame method, which is constructed by a Ceyear AV3672E vector network analyzer, as shown in Figure 6.

**Figure 7:**
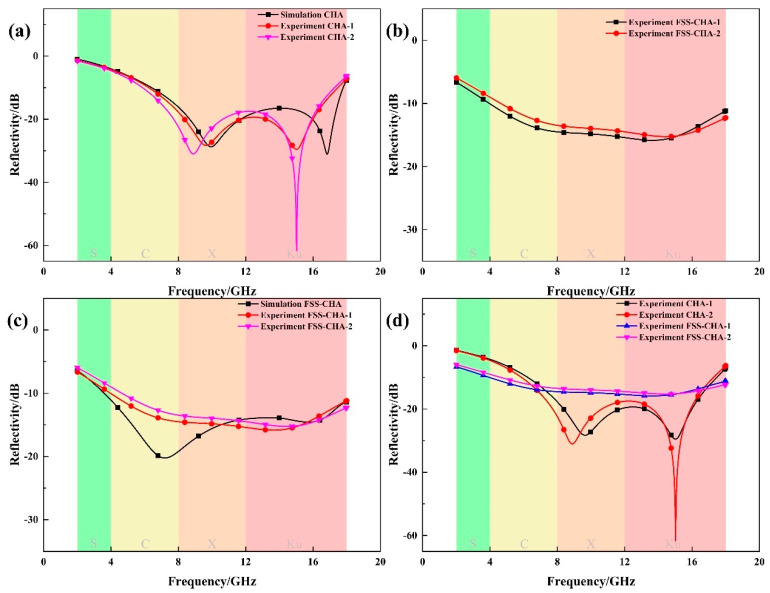
Simulation and experimental results of CHAs and FSS-CHAs. (**a**) Simulation and experimental comparison of CHAs, (**b**) reflection loss of two FSS-CHAs, (**c**) simulation and experimental comparison of FSS-CHAs, and (**d**) comparison of reflection loss between CHAs and FSS-CHAs.

To ensure the accuracy of the experiment, two CHAs and FSS-CHAs with the same parameters are prepared for electromagnetic performance testing, as shown in Figure 7.

In Figure 7a, it can be seen that the test results of the two CHAs are well discretized, and there are some gaps when compared with the simulation results, which are due to manufacturing errors. For example, when embedding the lock, it will touch the resistor sheet, causing the resistance of this part to increase. The results of the two FSS-CHAs tests are shown in Figure 7b, and it can be seen that the two test results are relatively close to each other. Figure 7c demonstrates the comparison between the FSS-CHA experimental results and the simulation results, and it can be seen that at high frequencies, the two are relatively similar, but at low frequencies, the results are not very good. This is because in addition to the fabrication error of CHA, the fabrication of FSS also produces errors, and also the fact that the composite of FSS and CHA requires the use of resin adhesive, which has unknown electromagnetic properties, and at the same time it cannot be simulated, which all contribute to the degradation of the results of the test. As shown in Figure 7d, combining FSS with CHA can effectively broaden the absorption bandwidth, and the electromagnetic wave absorption effect is significantly enhanced. Compared with the previously fabricated CHA, the effective absorption bandwidth (RL < −10 dB) is enhanced from the original 6.25–17.47 GHz with a bandwidth of 11.22 GHz to 3.96–18 GHz with a bandwidth of 14.04 GHz, an enhancement of 25.13%. In addition, not only electromagnetic waves at resonant frequencies, but electromagnetic waves at all frequencies are affected by the FSS, forming induced currents around the resistor sheet, thus consuming some of the energy of the electromagnetic wave, only around resonant frequencies this phenomenon is greater. This is why the absorption band increases but the peak value decreases in FSS-CHA compared to CHA. Excellent absorption results can be attributed to good impedance matching, dielectric loss, ohmic loss, etc. The absorbing materials for CHA and FSS-CHA are printed with conductive carbon ink, so the main loss is ohmic loss.

**Figure 8:**
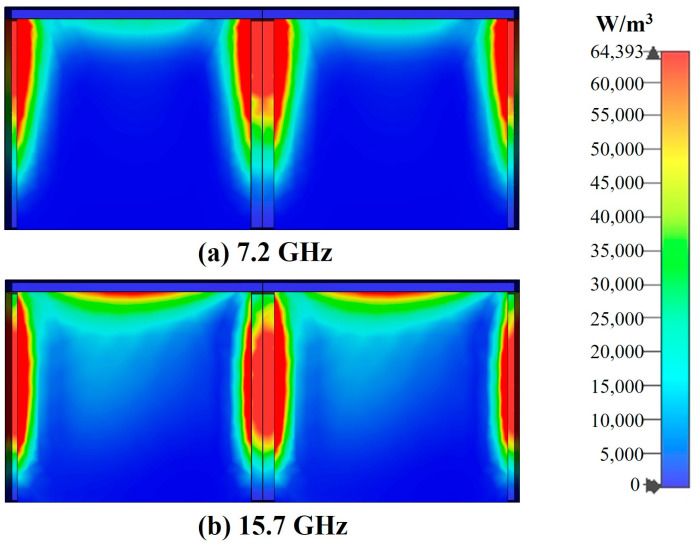
Power loss density distribution of FSS-CHA. (**a**) 7.2GHz, (**b**) 15.7GHz.

**Figure 9:**
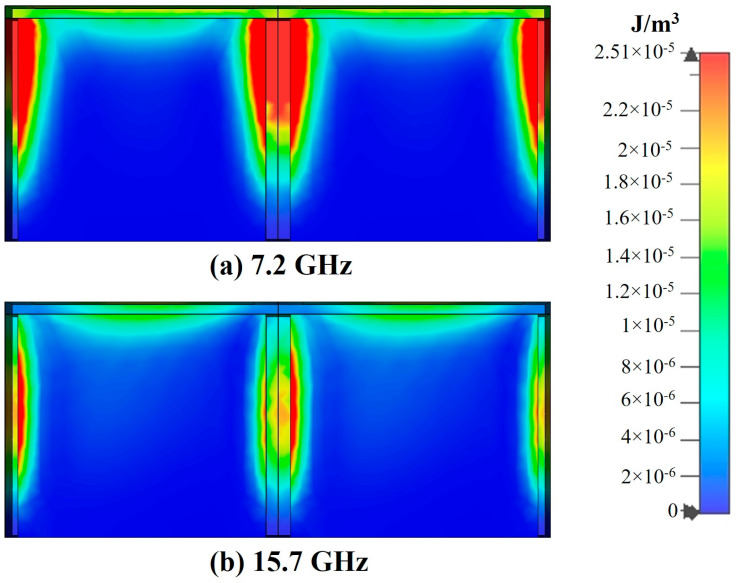
Electric field energy density distribution of FSS-CHA. (**a**) 7.2GHz, (**b**) 15.7GHz.

To investigate the mechanism of electromagnetic wave absorption, field monitors are set up at the frequencies *f*_1_ = 7.2 GHz and *f*_2_ = 15.7 GHz corresponding to the absorption peaks using CST EM Studio and the corresponding electric field energy density and power loss density clouds are extracted. As shown in Figure 8 and Figure 9.

At 7.2 GHz, in the FSS-CHA structure, there is a small power loss in the FSS and the main loss occurs in the upper layers and edges of the CHA. Meanwhile the distribution of electric field energy is the same as the power loss. This indicates that the absorption of FSS-CHA at low frequencies mainly comes from the dielectric loss of CHA and the edge diffraction effect of the periodic structure. At 15.7 GHz, there is a strong power loss in the FSS-CHA, as well as in the middle and edges of the CHA. Combined with the analysis of the distribution of the electric field energy, it can be inferred that the absorption of the FSS-CHA at high frequencies mainly comes from the resonance of the FSSs and the dielectric loss and diffraction effects of the CHA. There is a significant difference in the absorption mechanism of FSS-CHA at low and high frequencies, which also shows that it is feasible to combine FSS and CHA to achieve better impedance matching and improve the absorption band.

### 5.2. Mechanical Properties

**Figure 10:**
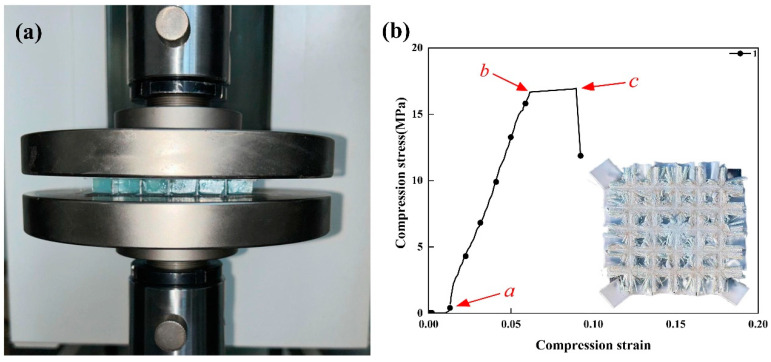
Compression properties of FSS-CHA. (**a**) Compression specimens of FSS-CHA, and (**b**) compression stress-compression strain curves of compression specimens.

To investigate the compressive properties of FSS-CHA, two specimens with the same parameters are prepared and tested for compression. Since the FSS has no load-bearing capacity, the specimens are prepared without mounting the metamaterial and only the PTFE skin is retained. Figure 10 shows the FSS-CHA and their compression stress-strain curves.

The damaged specimens are shown in Figure 10b. From the beginning of the loading stress to point a is the first stage, the stress increases with strain but is not uniform. This is because the PTFE surface layer is soft, the surface is not horizontal, and the stress is not uniform. After the stress is uniform, it enters the second stage, from a to b, the elastic stage, where the stress increases rapidly with the strain until the maximum load is reached. When the maximum load point b is reached, the soft PTFE is gradually cut off by the honeycomb core, so the stress does not increase in the section from b to c, but the strain increases. Meanwhile, the thickness of PTFE is 0.5 mm, which accounts for 0.05 of the overall strain, almost the length of the strain in the section from b to c. The brittle fracture occurs after point c, and the stress decreases. The ultimate compressive stress of the first specimen is 17.10 MPa.

### 5.3. Cost Statistics

In addition to excellent radar absorption and compressive properties, FSS-CHA is very cost effective. Statistically, the material cost of one sample of FSS-CHA (180 mm × 180 mm) is only USD 8.77, and the cost per square meter of FSS-CHA is only USD 270.7. As shown in Table 2, FSS-CHA has significant advantages in terms of wave-absorbing bandwidth and manufacturing cost.

The above unit prices are taken from the websites of the companies mentioned in the references, costs not mentioned are not accounted for.

## 6. Conclusions

Aiming at the problems of narrow frequency band of wave absorber and poor mechanical properties of the current wave absorber, a broadband wave absorber with excellent mechanical properties and low cost of FSS-CHA is designed and prepared by combining FSS and CHA. The specific conclusions are as follows:

1—The absorption tests show that the effective electromagnetic wave absorption range (RL < −10 dB) of FSS-CHA is 3.96–18 GHz, which is 25.13% higher than that of CHA. After simulation and analysis, this is due to the different absorption mechanisms at high and low frequencies.

2—Mechanical tests showed that FSS-CHA has excellent compressive properties with a compressive strength of 17.10 MPa.

3—The cost of FSS-CHA is low. After statistics, the cost of a sample of FSS-CHA (180 mm × 180 mm) is USD 8.77, which is USD 270.7/m^2^, which is much lower than that of other wave-absorbing materials.

FSS-CHA provides a new method to broaden the frequency band of wave-absorbing materials. The wide absorption band of FSS-CHA, along with its mechanical properties and low cost, is expected to provide a feasible solution for the application of wave-absorbing materials for ship masts.

However, this absorber is not perfect. In the relatively low-frequency band S-band (2–4 GHz), this absorber does not have a good absorption ability. At the same time, the compression strength needs to be further improved. For example, the impedance matching in the low-frequency band can be optimized by multilayer FSS to improve the absorption effect in the S-band. The honeycomb core can be made using materials with higher strength and lower dielectric constant, thus improving the compression strength without affecting the absorption ability. The conductive ink can be made using different kinds of polymers materials with multiple loss mechanisms to improve the absorption effect.

## Figures and Tables

**Table 1:** The parameters of the structure and their meanings.

Parameter Meanings	Values
FSS-CHA single cell cycle *p* (mm)	15
CHA wall thickness *d* (mm)	1
CHA height *h* (mm)	9
CHA wall resistance *r_f_* (Ω)	400
Resistance of each resistor sheet in the FSS *r*_1_ (Ω)	600
Cycles per resistor sheet in FSS *l* (mm)	9
PTFE thickness *h*_1_ (mm)	0.5
Carbon fiber back plate thickness *h*_2_ (mm)	0.4

**Table 2:** Comparison of FSS-CHA with previous wave absorbing materials.

Materials	Height (mm)	Absorption Domain (GHz)	Bandwidths (GHz)	Compressive Strength (MPa)	Costs (USD /m^2^)	Ref.
Graphene	3.35	7–18	11	-	96,590 ^a^	[31]
QFRP, resistive sheet	10	5.4–18	12.6	24.0	-	[28]
QFRC, carbon fiber, Resistive sheet, PMI	22	2.6–4, 6.6–40	34.8	5.80	>13,979 ^b^	[29]
FeSiAl, graphene, PLA	9	5.25–18	12.75	-	>4703 ^c^	[32]
Conductive carbon black	9	4.9–18	13.1	-	>1588 ^d^	[22]
FR-4, resistive sheet	9.9	3.96–18	14.04	17.10	270.7	This work

“-” represents no data available. PLA stands for polylactic acid. ^a^ The price of one layer of graphene is USD 48,475/m^2^, in the reference it is a double layer and can be found at www.graphene-supermarket.com [33]. ^b^ The unit price of QFRC prepreg is USD 75.9/m^2^. Extrapolating from the [21], 16.13 m^2^ of QFRC prepreg is required per m^2^ of 3D-RACHGS. The unit price for the resistive sheet is USD 2342.8/m^2^, and 5.38 m^2^ of the resistive sheet is required per m^2^ of 3D-RACHGS. The cost of materials for the production of screen-printed stencils is USD 150.4. ^c^ The specific surface area of graphene given in [22] is USD 620.24 m^2^/g, and the price of similar graphene in www.graphene-supermarket.com is USD 152.4/75 mg. According to the reference, at least 2.3 g of graphene is needed for 1 m^2^. ^d^ The unit price of the conductive carbon black is USD 6.35/g. It is inferred from [15] that at least 10 g of conductive carbon black is added to each sample (200 mm × 200 mm) and at least 250 g/m^2^.

## Data Availability

The original contributions presented in the study are included in the article, further inquiries can be directed to the corresponding author.
